# Ictal cardiovascular autonomic dysfunction during focal seizures induced by direct electrical stimulation: An observational study research protocol

**DOI:** 10.1371/journal.pone.0320357

**Published:** 2025-03-31

**Authors:** Dmitry Zhuravlev, Anna Marchenko, Anastasia Skalnaya, Marina Lebedeva, Igor Trifonov, Flora Rider, Nikolay Ierusalimsky, Sabir Burkitbaev, Natalia Semenovykh, Roman Luzin, Mikhail Sinkin, Vladimir Krylov, Alla Guekht

**Affiliations:** 1 Department of Neurology, Moscow Research and Clinical Center for Neuropsychiatry, Moscow, Russia; 2 Department of Neurology, Pirogov Russian National Research Medical University, Moscow, Russia; 3 Department of Neurosurgery, Russian University of Medicine, Moscow, Russia; 4 Department of Neurosurgery, Sklifosovsky Research Institute for Emergency Medicine, Moscow, Russia; 5 Technobiomed, Russian University of Medicine, Moscow, Russia; 6 Innovation Department, Institute of General Pathology and Pathophysiology, Moscow, Russia; 7 Institute of Higher Nervous Activity and Neurophysiology, Russian Academy of Sciences, Moscow, Russia; 8 Department of Medical Neurotechology, Pirogov Russian National Research Medical University, Moscow, Russia; Jikei University School of Medicine, JAPAN

## Abstract

**Introduction:**

Autonomic symptoms, such as changes in heart rate, blood pressure, or respiration often accompany epileptic seizures and, in some cases, may be life threatening or even contribute to sudden death. However, autonomic changes during seizures with onset from certain brain areas are insufficiently understood. Intracranial direct electrical stimulation during stereoelectroencephalographic (SEEG) monitoring in surgical candidates allows researchers to investigate autonomic responses to induced seizures in conscious patients with known precise location of the electrodes. We aimed to identify the epileptogenic focus locations or brain structures associated with ictal cardiovascular autonomic dysfunction during focal seizures induced by direct electrical stimulation.

**Methods and analysis:**

This is an observational study. In focal epilepsy patients undergoing presurgical evaluation with implanted intracranial SEEG electrodes, we will record heart rate (HR), beat-to-beat blood pressure (BP), and respiratory rate during the SEEG monitoring and stimulation conducted in accordance with the clinical needs. Tachycardia (HR > 100 bpm), bradycardia (HR < 60 bpm), hypertension (systolic or diastolic BP ≥ 140/90 mmHg), and hypotension (systolic or diastolic BP < 90/60 mmHg) during the first minute of induced clinical seizures will be considered as ictal cardiovascular autonomic dysfunction. We will use the chi-squared test to compare percentage of dysautonomia-associated seizures in the total number of induced seizures between cortical areas related to or interconnected with the central autonomic network and other cortical areas. Significance will be assumed for p-values < 0.05. At the time of submission, this study has enrolled thirteen patients and still on-going.

## Introduction

### Background

Autonomic symptoms (i.e., changes in heart rate, blood pressure, respiration, or electrodermal activity) often accompany epileptic seizures and are most prominent during tonic-clonic seizures with both focal or generalized onset [1–3]. However, life-threatening ictal autonomic dysfunction, such as bradyarrhythmia or central apnea, is observed only during focal-onset seizures, and notably these seizures are nonconvulsive in the majority of cases [4–6]. Also, ictal autonomic dysfunction indicates the involvement of the central autonomic areas in the epileptic activity and thus may be useful in the epilepsy diagnostics as well as in automatic detection of seizures, e.g., using wearable devices [7,8].

Brain structures directly involved in autonomic regulation of the human body form the central autonomic network (CAN) that includes insula, medial prefrontal cortex, amygdala, hypothalamus, periaqueductal grey matter, parabrachial complex of the pons, solitary nucleus, and ventrolateral reticular formation [9]. Direct electrical stimulation of the cortical CAN structures causes changes in respiration, blood pressure, and heart rate [10,11]. Thus, the fundamental study of Oppenheimer and colleagues (1992) demonstrated that intraoperative electrical stimulation of the left insular cortex led to bradycardia and depressor vascular responses whereas stimulation of the right insular cortex resulted in tachycardia and pressor responses [11].

Intracranial electrical stimulation during stereoelectroencephalographic (stereo-EEG, SEEG) monitoring in surgical candidates allows researchers to investigate responses in conscious sedation-free patients with known and visualized precise location of the electrodes. Moreover, stimulation during SEEG commonly induces nonconvulsive seizures that makes possible to monitor ictal changes of heart rate and blood pressure.

Sanchez-Larsen et al. (2021, 2024) reported hypotensive responses to direct electrical stimulation of the left insula, hypertensive responses to stimulation of the right insula, a decrease in heart rate and cardiac output and an increase in stroke volume during electrical stimulation of the right as well as left insula [12,13]. Lacuey et al. (2018) recorded systolic hypotensive changes as a result of stimulation of subcallosal neocortex (Brodmann area 25) in 4 of 12 patients [14]. Inman et al. (2020) described decelerations of heart rate and increase in electrodermal activity during the amygdala stimulation [15].

All the above studies described autonomic responses to the intracranial stimulation of the presumably intact brain structures during SEEG monitoring as a part of presurgical evaluation in patients with drug-resistant epilepsy, i.e., evaluated effects of the activation of the cortical areas which were not supposed to be involved in epileptogenesis in these patients.

In opposite to physiological autonomic modulation, autonomic changes during epileptic seizures are less understood. For example, epileptic activity in areas that are not considered as CAN structures but are closely interconnected with them, e.g., in hippocampus or other limbic and paralimbic structures, may result in ictal autonomic dysfunction as well [16,17].

A few papers described ictal autonomic changes that accompanied focal onset seizures recorded during SEEG monitoring. In the study by Chouchou et al. (2017), ictal tachycardia was associated with epileptic activity (high frequency oscillations) involving amygdala and anterior hippocampus, independent of the seizure lateralization, but not insula (9 patients, 37 seizures) [18]. Pensel et al. (2021) also recorded ictal tachycardia during hippocampal seizures in both hemispheres with more prominent decrease of PR intervals in seizures with left-sided onset (14 patients, 56 seizures) [19]. The role of other cortical structures in ictal arrhythmias supported by the SEEG recordings is largely unknown. Furthermore, there is a lack of studies evaluating blood pressure and baroreflex sensitivity changes during focal-onset seizures related to certain cortical structures.

### Hypothesis

Epileptic activity during seizures induced by stimulation of the CAN structures and structures closely interconnected with CAN causes ictal cardiovascular autonomic dysfunction.

### Objective

Primary objective

1) To identify the epileptogenic focus locations or brain structures associated with ictal autonomic cardiovascular dysfunction during focal seizures induced by direct electrical stimulation

Secondary objectives

2) To verify whether autonomic dysfunction is associated with: (i) stimulation of certain brain areas, or (ii) induced epileptic seizure activity in the areas, or (iii) propagation of epileptic activity to interconnected areas
3) To verify whether seizures induced from the CAN structures and structures closely interconnected with CAN are associated with postictal baroreflex changes.

## Materials and methods

### Study setting and key personnel

This observational study will be conducted in neurosurgery departments of Russian University of Medicine and Sklifosovsky Research Institute for Emergency Medicine located in Moscow, Russian Federation. Data interpretation and analysis will be provided by neurology and radiology departments of Moscow Research and Clinical Center for Neuropsychiatry. The study team is a multidisciplinary team of clinicians and researchers including neurologists, neurophysiologists, neurosurgeons, radiologists, and physiologists. The key personnel are specialized in epilepsy care and autonomic nervous system.

### Study design and population

**Fig 1:**
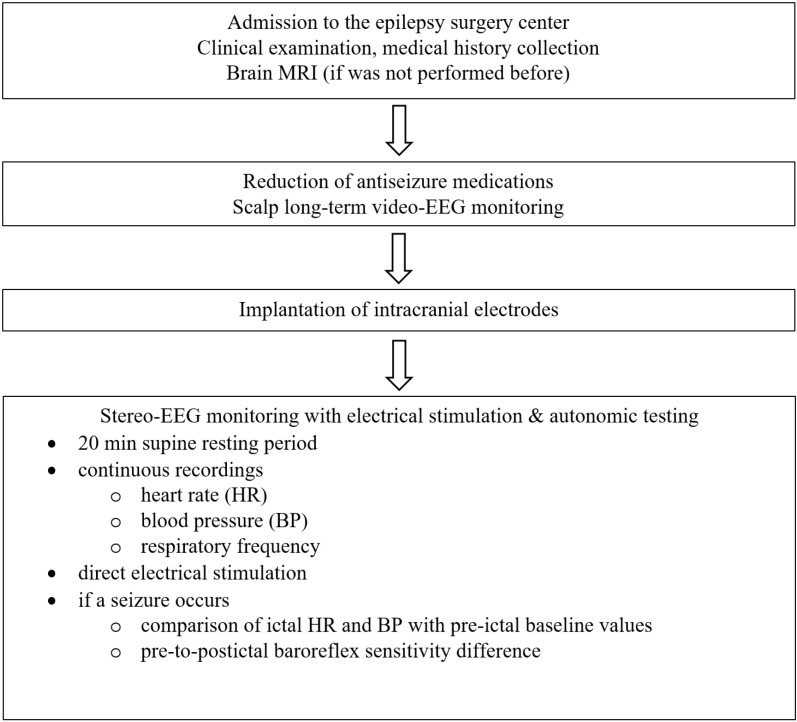
The project design scheme. This figure reflects all the steps of participants evaluation and data collection.

We will enroll patients referred to our centers for epilepsy surgery who will undergo pre-surgical evaluation including SEEG and direct electrical stimulation of the suspected brain structures in order to identify seizure onset zone (see Fig 1). All the procedures will be dictated by clinical needs only.

Inclusion criteria:

- age between 18 and 55 years;
- drug-resistant focal epilepsy [20] with the following structural etiologies: hippocampal sclerosis, focal cortical dysplasia, heterotopy, polymicrogyria, long-term epilepsy associated tumors (LEAT), and MRI-negative forms of focal epilepsy;
- implanted intracranial SEEG electrodes and direct electrical stimulation of suspected epileptic brain regions;
- clinical seizures elicited by cortical stimulation and recorded during SEEG.

Exclusion criteria:

- the following MRI abnormalities: vascular abnormalities (ischemic or hemorrhagic infarction, developmental venous anomalies, cavernous malformations), traumatic brain injuries, tuberous sclerosis, inflammation-related MRI findings (remote neurocysticercosis, remote herpes encephalitis, remote autoimmune encephalitis), non-epileptogenic MRI findings (hippocampal incomplete inversion, arachnoid or epidermoid cyst);
- clinically manifested somatic (e.g., arterial hyper- or hypotension, arrhythmias, diabetes melitus), psychotic or comorbid neurological disorders (e.g., multiple sclerosis, neurodegenerative diseases, neuromuscular disorders, cerebral palsy);
- patients with severe autonomic dysfunction;
- history of substance abuse;
- use of medications affecting autonomic cardiovascular regulation except antiseizure medications.

Written informed consent will be obtained from all participants prior to the study. Any participant may be excluded from the study by the own will without any explanation.

### Clinical, neuroimaging and neurophysiological data

We will perform clinical examination and evaluate medical records of all participants to estimate clinical characteristics of epilepsy including its form and etiology, types, semiology and frequency of seizures, duration of the disease, and treatment history. After admission to the epilepsy surgery centers regimen of antiseizure medications will be modified according to the diagnostic needs, usually medications are temporally (for the evaluation period) reduced or discontinued.

All admitted patients will undergo 1.5 T or 3.0 T brain magnetic resonance imaging (MRI) and long-term video-EEG monitoring in accordance with the international standards [21,22]. We will include in the study only those patients who will undergo implantation of intracranial SEEG electrodes and electrical stimulation based on the clinical need, e.g., when noninvasive data will be conflicting or inconclusive or when epileptogenic zone will be proximal to eloquent cortex.

Neurosurgeons will implant “The AD-TECH Depth Electrodes” (or similar, length 2.41 mm/diameter 1.10 mm with eight or six number of contacts) using optic navigation in the brain regions suspected to be involved in seizure onset and early propagation of the expected ictal pattern. Radiologists will define the precise anatomical location of the electrodes through modeling of preimplantation MRI with postimplantation computer tomography (CT) images. MPR Axial T1 sequences with isotropic voxel will be used to obtain anatomic data, particularly to segment and mask cortical and subcortical brain structures using Freesurfer 7.4.1 software (https://surfer.nmr.mgh.harvard.edu/) [23]. CT images will be coregistered with MRI on the MNI 152 template using FMRIB’s linear image registration tool (FLIRT, https://fsl.fmrib.ox.ac.uk/fsl/). Finally, CT images and masks of the brain structures segmented by MRI will be matched using Freesurfer Freeview visualization tool.

### Direct electrical stimulation

In order to evoke a habitual seizure and thus to confirm the suspected seizure onset zone, electrical stimulations will be provided under continuous video-EEG control (BE Plus LTM, EB Neuro S.p.A., Italy, or similar) using Nicolet Cortical Stimulator (Natus, USA, or similar). The stimulations will be performed by a trained neurophysiologist following the standards of clinical practice [24,25]. Bedside direct cortical stimulation will be carried out between contiguous contacts at various levels of the electrode axis using sustained trains of biphasic current pulses with pulse width 0,3 or 1,0 msec, intensity increasing (unless a seizure occurs) from 1 mA to 6 mA depending on the pulse width in order to maintain approximate equivalence in the charge density, at 50 Hz frequency for 3 seconds per train. If a clinical seizure occurs at low intensities of the stimuli, then electrical stimulation of these contacts will be capped without increasing of parameters. The time and parameters of each stimulation will be noted.

We will define the habitual stimulation-induced seizure as stimulation-induced subjective or objective clinical signs, previously described by the patient and/or witnessed, and/or previously observed during spontaneous seizure recordings, accompanied by ictal epileptic activity on EEG [26]. Non-habitual clinical stimulation-induced seizures will be also analyzed separately. Ictal and postictal testing will be performed in case an electroclinical seizure is detected [27]. The detailed description of seizure semiology will be collected for every seizure.

During the process of stimulation at least two persons of healthcare personnel (neurologist and neurophysiologist) will be present to monitor autonomic parameters and EEG and to intervene in the case of a seizure. During electrical stimulation sessions patients will be asked to perform simple tasks such as pictures naming or reading that will help with immediate recognition of even subtle clinical signs. After each stimulation patients will be asked if they feel anything in particular and if so to describe it.

In order not to provoke misleading feelings or symptoms due to increased level of anxiety, the exact moment of stimulation will not be disclosed to the patients.

### Autonomic parameters during electrical stimulation

All participants will be lying supine for at least 20 minutes prior to stimulation to assure a stable cardiovascular resting state. During at least 5 minutes before the start of the stimulation procedure (to assess initial values of cardiovascular autonomic parameters) and during the stimulation sessions we will continuously record heart rate as electrocardiographic (ECG) RR-intervals (RRIs) using single lead chest recording, beat-to-beat blood pressure (BP) using non-invasive finger-pulse photoplethysmography [28], respiratory frequency by means of a nasal thermistor sensor or rheopneumogram, with sampling rates of 1000 Hz (Spiroarteriocardiorhythmograph, Incart, Saint-Petersburg, Russian Federation). The software locates R-peaks by proprietary algorithm, checks RRI time series for artifacts and arrhythmic events and allows an investigator to check the located R-peaks visually and to correct them manually if indicated. Beat-to-beat blood pressure is adjusted to the arm blood pressure every three to six minutes measured by automated auscultative and oscillometric methods.

To evaluate arterial baroreflex sensitivity (BRS), we will use the sequence method. Spontaneously occurring sequences of synchronized changes in BPsys and RRIs will be automatically detected. Increases as well as decreases of BPsys ≥ 1 mmHg per heart beat occurring together with at least three concurrent increases respectively decreases in RRIs ≥ 4 msec per hear beat will be considered as one sequence. For each identified sequence, a regression analysis will be performed to calculate the slope of the linear relationship between changes in BPsys and RRIs. The average of the slopes in the detected sequences will be considered as the overall baroreflex sensitivity.

The moment of stimulation will be marked on autonomic and EEG traces. Pattern of a seizure will be carefully evaluated.

For every clinical seizure, recordings during at least one minute before the stimulation that induces the seizure will be considered as a baseline for autonomic cardiovascular parameters. Changes of heart rate and blood pressure during one minute following the stimulation that provokes ictal EEG pattern will be considered as clinically significant ictal autonomic dysfunction when all of the following criteria are met:

- at least one of the parameters reaches clinically significant thresholds: (i) heart rate > 100 bpm (considered as tachycardia), (ii) heart rate < 60 bpm (considered as bradycardia), (iii) systolic or diastolic blood pressure ≥ 140 or ≥ 90 mmHg respectively (considered as arterial hypertension), (iv) systolic or diastolic blood pressure < 90 or < 60 mmHg respectively (considered as arterial hypotension);
- heart rate or blood pressure did not reach relevant clinically significant threshold during prestimulation baseline period;
- heart rate or blood pressure increases or decreases for more than three-fold standard deviation from the mean baseline values during the first minute of the seizure.

If stimulation induces electrographic seizure or autonomic changes without epileptiform activity, the stimulation will be discontinued and we will wait until heart rate and blood pressure return to their baseline values and at least two minutes after the end of the ictal pattern before the next stimulation.

Data on the ictal autonomic changes as well as on the absence of the changes will be collected for every induced seizure and site of stimulation including duration of autonomic changes and seizures and areas of the seizure propagation. Additionally, we will collect BRS values during one-minute postictal period and during one-minute pre-ictal stimulation-free period for every induced seizure.

Brain areas associated and not associated with ictal autonomic dysfunction will be indicated on the map of the brain structures and on individual MRI of the patients. The dysautonomia-associated brain areas of seizure onset and seizure propagation will be indicated and analyzed separately.

### Statistical analysis

For statistical calculations we will use IBM SPSS Statistics version 23.

We will use the Kolmogorov-Smirnov test or the Shapiro-Wilk test (if the number of samples <  50) to test data for normal distribution. Normally distributed data will be presented as mean ±  standard deviation (SD), non-normally distributed data will be presented as median with interquartile range (Q1; Q3).

To test our hypothesis, we will use the chi-squared test to compare percentage of dysautonomia-associated seizures in the total number of induced clinical seizures between the two groups of brain areas: (i) cortical structures related to or interconnected with CAN (medial temporal lobe structures, medial prefrontal cortex, insular cortex) and (ii) other brain areas (lateral temporal cortex, lateral frontal cortex, parietal cortex, occipital cortex). Additionally, to describe brain areas associated with ictal cardiovascular autonomic dysfunction, we will calculate the percentage of dysautonomia-associated seizures in the total number of induced clinical seizures for all of the cortical areas (separately for left and right hemisphere): medial temporal cortex, medial prefrontal cortex, insular cortex, lateral temporal cortex, lateral frontal cortex, parietal cortex, occipital cortex.

To verify whether seizures induced from the structures related to or closely interconnected with CAN are associated with postictal baroreflex changes, we will compare pre- and postictal values of BRS for the two groups of the brain areas described above using paired Student t-test in case on normally distributed data and Wilcoxon signed-rank test in case of non-normally distributed data.

Significance will be assumed for p-values < 0.05.

### Data management

Clinical data will be anonymized and stored on password-protected and encrypted computers to maximize confidentiality and security. All paper files are kept in a secure, locked location accessible only to the research team. All scientific data will be stored within the research center protected data store with access for the research team members only.

### Ethics and dissemination

The study was approved by the ethical committee of Moscow Research and Clinical Center for Neuropsychiatry (approval code № 76, 19 January 2024). The stimulation will be provided according to the clinical needs only. Written informed consent will be obtained from all participants prior to the study. Any participant may be excluded from the study by the own will without any explanation. The results will be submitted for presentation at the international neurological meetings and publication in specialized medical journals. Also, patients’ communities will be involved in the dissemination of the results of the study, if appropriate.

### Safety considerations

This study is observational, with no direct risks associated with participation. If a stimulation induces a focal-to-bilateral tonic-clonic seizure, the procedure will be discontinued. In case of spontaneous seizure occurrence during a stimulation session neurologist provides all safety considerations and clinical testing of awareness according to the ILAE recommendations [27]. If a seizure will last more than five minutes then, according to the National Association of Epilepsy Centers recommendations [22], we will use intravenous or non-intravenous emergency antiseizure medications (in our centers – Diazepam) and transfer a patient to the intensive care unit.

### Study timeline and status

This is an observational study scheduled for 19 January 2024–19 February 2026. Study enrollment began on February 2024. At the time of submission, this study has enrolled thirteen patients and still on-going.

### Patient and public involvement

We consulted with the patient community regarding appropriate terminology that will be used during the study and in the publication of its results. We are planning to involve patients’ communities in the dissemination of the study results.

## Discussion

### Strengths and limitations of the study design

To our knowledge, it is the first study to address ictal blood pressure and baroreflex changes accompanying induced seizures during SEEG monitoring. According to our experience, the majority of induced seizures are non-convulsive, that will allow us to obtain artifact-free heart rate and beat-to-beat blood pressure recordings before, during, and after a seizure. During stimulation, patients with implanted intracranial electrodes are conscious and thus will be able to describe, beside objective signs, semiology of induced seizures if awareness is not impaired. Accurate neuroimaging data will allow us to define the precise seizure onset zone. Stereo EEG monitoring will allow us to confidently exclude nonepileptic events and provide us with information regarding propagation of epileptic activity. Consequently, we will be able to convincingly describe the association between ictal cardiovascular autonomic dysfunction and certain brain areas involved in the seizure generation and propagation.

Nevertheless, our study design has several limitations. First, the exact location of implanted intracranial electrodes depends on individual electroclinical hypothesis. While some structures are commonly investigated, e.g., medial temporal structures, other areas of interest such as certain neocortical sites are highly variable. Therefore, we expect to see imbalance in the number of recorded seizures regarding different brain areas. Hopefully, our main research interest is focused on the CAN structures, and several key cortical CAN structures and structures that are closely interconnected with them, e.g., amygdala and hippocampus, are the most common brain structures to be investigated during SEEG monitoring and stimulation.

Second, as soon as the number of deep brain electrodes and their location are limited to the individual clinical need, we will be able to record the propagation of epileptic activity only within the individual set of the electrodes and some areas of interest may remain uncovered. Still, deep brain electrodes are usually implanted in the structures which are highly likely to be involved in the seizure generation or propagation and, accordingly, we believe the obtainable data will match our research objectives.

Third, many people with epilepsy undergoing presurgical evaluation with direct electrical stimulation are taking antiseizure medications that may potentially influence the autonomic regulation and response to the stimulation. Nevertheless, the medications are commonly reduced or, in some cases, even temporally discontinued during the evaluation that also should mitigate their effect on the autonomic nervous system.

### Participants

Participants of our study are individuals with focal epilepsy undergoing presurgical evaluation that includes diagnostic direct electrical stimulation of the brain structures. On the one hand, this cohort of people have the most severe, drug resistant form of epilepsy, sometimes with multiple structural abnormalities or epileptogenic foci. Consequently, the results of our study could not be interpolated to the whole population of people living with epilepsy, particularly to those with generalized or well controlled epilepsy. On the other hand, surgical candidates are the most vulnerable cohort of people living with epilepsy in terms of the high risk of the sudden unexpected death (SUDEP) [29], and revealing the mechanisms of ictal cardiovascular autonomic dysfunction is crucial for this population.

### Sample size of the study population

We will calculate the percentage of seizures which are associated with ictal cardiovascular autonomic dysfunction in the total number of induced seizures. Only clinical seizures will be analyzed. When a clinical seizure is induced in a particular brain site of a participant, we will stop the stimulation in that site meaning that the number of the analyzed seizures will be equal to the number of the participants for each of the studied brain areas.

Considering unpredictable set of the brain structures where seizures could be potentially induced, the exact number of the seizures per each cortical area, and proportion of dysautonomia-associated seizures due to interindividual variability of the clinical manifestation of epilepsy, reasonable calculation of the optimal sample size for our study is challenging. After consulting with a biostatistician, we concluded that our objectives and study design do not imply an accurate sample size calculation for our methodology. According to the literature data, similar descriptive studies are carried out with 10 to 14 patients [12–14]. Based on the previous studies reporting that ictal tachycardia, which is the most studied ictal cardiovascular autonomic dysfunction, can be recorded in 62–77% of the temporal lobe seizures and in 11–22% of the extratemporal seizures [30], we can also expect that 12–13 patients should be sufficient for the qualitative comparative analysis between the two groups with 80% statistical power. Therefore, based on our best assumptions about the study sample size that would be sufficient and feasible, our team decided to include in the study at least 15 patients to each of the two groups.

### Criteria of cardiovascular autonomic dysfunction

To our best knowledge, there is no commonly recognized specific criteria for ictal cardiovascular autonomic dysfunction. Considering autonomic dysfunction diagnosed by the standardized cardiovascular tests in people with autonomic failure, classic orthostatic hypotension is diagnosed when systolic BP decreases by ≥ 20 mmHg or diastolic BP decreases by ≥ 10 mmHg and initial orthostatic hypotension is diagnosed when systolic BP decreases by ≥ 40 mmHg or diastolic BP decreases by ≥ 20 mmHg [31,32]. Postural orthostatic tachycardia can be diagnosed when heart rate increases by 30 bpm (or by 40 bpm for individuals 12–19 years) [31,32]. However, mechanisms underlying autonomic dysfunction in patient with autonomic failure and during a seizure may differ.

In some wearable devices designed for people with epilepsy to detect epileptic seizures, increase of heart rate by 50 bpm is considered as a threshold for significant ictal tachycardia [33–35]. At the same time, wearable devices use multimodal analysis for automated seizure detection and are best suitable for tonic-clonic seizures and seizures with prominent tachycardia [33–35]. We considered changes of heart rate of 50 bpm to be too strict criteria for our study design and objectives. Furthermore, all the above criteria for significant heart rate changes are suitable to diagnose tachycardia but not bradycardia.

Some authors use general (cardiological) criteria defining tachycardia as HR > 100 bpm, bradycardia as HR < 60 bpm, hypertension as systolic BP ≥ 140 mmHg and/or diastolic BP ≥ 90 mmHg, and hypotension as systolic BP < 90 mmHg and/or diastolic BP < 60 mmHg, [36–38]. This approach seems to be imperfect keeping in mind that some people have initial individual tendency to hyper- or hypotension as well as tachy- or bradycardia at baseline. However, people who have increased or decreased blood pressure or heart rate before the start of the stimulation procedure are highly likely to have initially compromised autonomic regulation and will be excluded from the study. We decided that aforementioned general criteria are best suited for the aim of our study as soon as they are easy to use, have clear clinical significance and ensure that ictal autonomic changes will be analyzed in people with normal baseline hemodynamic parameters. We will consider the changes as significant only if in the prestimulation one minute period heart rate and blood pressure did not reach these general thresholds. Such approach will additionally allow us to exclude from the analysis episodes when heart rate or blood pressure may increase during the stimulation procedure due to anxiety or tiredness. To make sure that the discussed ictal changes of heart rate or blood pressure are not accidental, we will include in analysis only those changes that exceed three-fold standard deviation thresholds from the mean prestimulation values as soon as these changes will exceed 99.73% of the baseline values.

### Accuracy of non-invasive beat-to-beat blood pressure measurements

According to the publications dedicated to seizure-related modulation of cardiovascular parameters, non-invasive beat-to-beat blood pressure measurement is considered to be the most preferable method for blood pressure measurement [36,37,39]. Some clinical protocols recommend this method in addition to video-EEG or SEEG recordings [40]. In contrast to arm-cuff periodic measurements, non-invasive recordings provide continuous monitoring of BP changes all over the procedure that is essential for long-lasting investigations. According to comparative studies, accuracy of non-invasive beat-to beat BP measurements is comparable to intraarterial invasive BP evaluation which is known as golden standard [41]. However, there are several significant limitations to this method that may be important for the following data analysis. Continuous non-invasive beat-to-beat BP monitoring may be quite sensitive to peripheral vascular resistance changes and demonstrates lower levels of BP values in comparison with arm-cuff periodic measurements [42]. Another possible problem is moving or breathing artifacts which may interfere with BP signal during seizures, particularly during focal-to-bilateral tonic-clonic seizures, and thus may lead to BP data exclusion from analysis [41].Nevertheless, according to our practice, the majority of seizures induced via direct electrical stimulation during SEEG monitoring are nonconvulsive.

### Origin of autonomic changes during stimulation-induced seizures

The nature of autonomic dysfunction in people living with epilepsy is not clearly verified yet. According to previous studies, autonomic manifestations of seizures are very common and may be clinically significant [43]. As an example, blood pressure and heart rate may increase to 30% and 55% respectively from their baseline levels [36,37,39]. Many studies described the presence of ictal hyper- or hypotension or heart rate abnormalities (ictal asystole, AV-conduction disturbance, etc) during ictal period [44]. It is hypothesized that ictal cardiovascular autonomic dysfunction is caused by disruption of the central autonomic network during seizures [43].

Cardiovascular autonomic changes are also recorded during direct electrical stimulation of certain cortical structures [11–15]. It may be challenging to distinguish autonomic changes related to the cortical stimulation from those related to the seizures. However, presence of autonomic changes during electrical stimulation are highly dependent on intensity of the stimulus, individual excitability of the structures, and brain region it was applied to [17,45–47]. Electrical stimulation is perceived as a trigger for epileptogenic network synchronization and its neurophysiological effects on the brain tissue may be presumably identical to a short focal seizure [24]. According to the previous studies, stimulation-induced autonomic changes were less prominent compared to the seizure-related ones and laid within the limits of physiological modulation [12,13]. In stimulation-induced seizures, both clinical signs, if present, and EEG discharges outlast the electrical stimulus and the intracranial EEG pattern evolves in frequency and distribution as is seen in spontaneous seizures. Therefore, according to the concept of electroclinical correlation [24], if autonomic changes occur after the onset of the ictal pattern, they can be considered as a semiological expression of the induced seizure. From that point, we propose that clinically significant changes of heart rate and blood pressure exceeding thresholds discussed above are more likely to be caused by a seizure rather than by a stimulation.
