# Correction: Ictal cardiovascular autonomic dysfunction during focal seizures induced by direct electrical stimulation: An observational study research protocol

**DOI:** 10.1371/journal.pone.0331935

**Published:** 2025-09-08

**Authors:** Dmitry Zhuravlev, Anna Marchenko, Anastasia Skalnaya, Marina Lebedeva, Igor Trifonov, Flora Rider, Nikolay Ierusalimsky, Sabir Burkitbaev, Natalia Semenovykh, Roman Luzin, Mikhail Sinkin, Vladimir Krylov, Alla Guekht

The first affiliation for the 12^th^ author is incorrect. The correct affiliation is not indicated. Vladimir Krylov is not affiliated with #2 but with: Department of Fundamental Neurosurgery, Pirogov Russian National Research Medical University, Moscow, Russia.
